# Factors associated to serum paraoxonase 1 activity in patients with cardiovascular disease

**DOI:** 10.20945/2359-3997000000354

**Published:** 2021-04-12

**Authors:** Aline Longo, Gabriel Barreto Veiga, Maria Isabel Schiavon Cousen, Caroline Karpinski, Augusto Schneider, Bernardete Weber, Eduardo Gehling Bertoldi, Lucia Rota Borges, Renata Torres Abib Bertacco

**Affiliations:** 1 Universidade Federal de Pelotas Faculdade de Nutrição Pelotas RS Brasil Faculdade de Nutrição, Universidade Federal de Pelotas, Pelotas, RS, Brasil; 2 Instituto de Pesquisa – Hospital do Coração São Paulo SP Brasil Instituto de Pesquisa – Hospital do Coração (IP – HCor), São Paulo, SP, Brasil; 3 Universidade Federal de Pelotas Faculdade de Medicina Pelotas RS Brasil Faculdade de Medicina, Universidade Federal de Pelotas, Pelotas, RS, Brasil

**Keywords:** PON1, atherosclerosis, antioxidants, genetics, diet

## Abstract

**Objectives:**

Paraoxonase 1 (PON1) is an enzyme that has antioxidant potential, which confers a protective effect against the atherosclerotic process. However, studies associating genetics, dietary patterns and PON1 activity in individuals with cardiovascular disease (CVD) are scarce. Thus, the aim of the current study was to evaluate the influence of dietary factors on serum PON1 in CVD patients.

**Subjects and methods:**

Cross-sectional, sub-study of the BALANCE Program Trial. All patients aged 45 years or older and had evidence of established atherosclerotic disease in the preceding 10 years. Body weight, height, waist circumference, blood pressure, lipid profile and fasting glucose were collected. Food intake was assessed with 24-h dietary recall. Data was analyzed using SAS University Edition and a P value ≤ 0.05 was considered statistically significant. Sample was divided into three groups, according to the PON1 T(-107)C genotype (CC, CT and TT) and serum PON1 activity (Low, Medium, High).

**Results:**

There were no genotype differences for major factors. However, the systolic blood pressure was lower for CT individuals (p<0.05). Intake of cholesterol, saturated fatty acids (SFA) and monounsaturated fatty acids (MUFAS) was higher in patients with lower PON1 activity. Lipid ingestion tended to be higher in patients with lower PON1 activity (p=0.08). In the multivariate logistic regression model, SFA intake (P=0.03), genotype (P=0.09), gender (P=0.04), age (P=0.07) and carbohydrate intake (P=0.16) contributed the most to the serum PON1 activity.

**Conclusion:**

Based on these findings, nutritional guidance for these patients becomes essential, since dietary components interact with serum PON1 activity more than genotype.

## INTRODUTION

The paraoxonases (PON) are a group of enzymes (1), encoded by genes on chromosome 7 (2). PON1 it is the most studied member of this family, can hydrolyze organophosphate compounds (3) and has antioxidant potential (4^,^^5^). PON1 is synthesized by the liver and released into the bloodstream associated with high density lipoprotein (HDL) molecules (6,7). PON1 has a protective effect against the atherosclerotic process as it inhibits oxidation of low-density lipoproteins (LDL) (8). In this context, more than 160 single nucleotide polymorphisms (SNPs) in the PON1 gene are known (9). One of the most significant polymorphisms is located in the promoter region of PON1, known as C(-107)T or rs705379, which accounts for 12% of the total variation in serum PON1 activity (10). The presence of this SNP affects liver gene expression and serum enzyme activity, and the presence of the C/G allele is associated with the twice as high PON1 serum activity compared to the presence of the T/A allele (11–13). Therefore, clearly demonstrating the important role of genetics in serum PON1 activity.

In addition to genetic factors, eating habits and the environment can also affect serum PON1 activity (11). Despite this, dietary habits contribute less than genetics to the total serum PON1 activity (10,14). Nevertheless, consumption of fatty acids and cholesterol can modulate serum PON1 activity (10,11,15). However, while some studies indicate that cholesterol is able to increase the serum PON1 activity (10), others have not observed the same association (12). In addition, the literature indicates that the effects of dietary intake on PON1 activity are dependent on PON1 genotypes, as diets rich in saturated fatty acids (SFA) decrease serum PON1 activity in PON1 -107 CC individuals only (11). Moreover, it is known that the serum PON1 activity is lower in CVD patients compared to healthy individuals (16).

Despite these evidence, studies associating genetics, dietary patterns and PON1 activity in individuals with CVD are scarce. In this context, the aim of the current study was to evaluate the influence of dietary factors on serum PON1activity in patients with CVD.

## SUBJECTS AND METHODS

### Volunteers and ethics

Volunteers are part of Brazilian Cardioprotective Nutritional Program Trial (BALANCE Program Trial) (17), which was being funded by Hospital do Coração (HCor) as part of the “*Hospitais de Excelência a Serviço do SUS* (PROADI-SUS) program”, in partnership with the Brazilian Ministry of Health. All eligibility criteria were reported on the study protocol (17).

The population considered for this cross-sectional sub-study consisted of 64 volunteers from one collaborating center in Southern Brazil (Pelotas, RS, Brazil). Data collected refer to the 12 months of follow-up of the original study. This sub-study was approved by the local ethics committee (CAAE number 48527415.3.0000.5317) and all participants provided written informed consent prior to inclusion.

All patients were aged 45 years or older and had evidence of established atherosclerosis disease in the preceding 10 years: (a) coronary disease (defined by previous myocardial infarction, stable or unstable angina, history of atherosclerotic stenosis ≥70% of the diameter of any coronary artery on conventional or computed tomographic (CT) coronary angiography, or history of angioplasty, stenting, or coronary artery bypass surgery); (b) previous stroke; (c) peripheral vascular disease (ankle/arm ratio <0.9 of systolic blood pressure in either leg at rest, angiography or Doppler demonstrating >70% stenosis in a cardiac artery, intermittent claudication, vascular surgery for atherosclerotic disease, amputation due to atherosclerotic disease, or aortic aneurysm). The exclusion criteria were: neurocognitive or psychiatric conditions; life expectancy less than 6 months; pregnancy or lactation; liver failure with a history of encephalopathy or anasarca; renal failure with indication for dialysis; congestive heart failure; previous organ transplantation; wheelchair use; or any restrictions to receiving an oral diet.

### Sociodemographic, clinical, and behavioral characteristics

Trained interviewers administered a structured questionnaire comprising questions on clinical characteristics, and blood pressure was obtained by a trained professional. All data were recorded in an electronic case report form (e-CRF).

Body weight and height were measured using a digital calibrated scale with a coupled stadiometer (Filizola^®^), with an accuracy of 0.1 kg and 0.1 cm, respectively. Waist circumference was obtained by inelastic tape measure, at midway between the lowest rib and the iliac crest using an anthropometric tape, with an accuracy of 0.1 cm. Body mass index (BMI) was calculated from weight (kg) divided by squared height (m).

### Laboratory measurement

All volunteers were fasted for at least 12 h (maximum 14 h) before phlebotomy. Total cholesterol, HDL cholesterol, triglycerides and glucose were determined by enzymatic colorimetric dry chemistry method (Ortho-Clinical Diagnostics VITROS 5.1), in venous blood, and LDL cholesterol was estimated using the Friedewald equation (18).

### Dietary assessment

Food intake data were obtained by 24-h dietary recalls and recorded in the Nutriquanti software (São Paulo, SP, Brazil), a Brazilian software which prioritizes the Brazilian composition food tables. A photo album containing images of standardized food portion sizes, specifically prepared by BALANCE Program Trial (17), was used to assist food intake assessment.

### Genotyping

For DNA extraction whole blood samples were used according to a validated protocol (19). To amplify the region where the SNP PON1 T (-107) is located, PCR was performed using 10 μL of GOTaq^®^ mixture (Promega, Madison, WI, USA), 1 μL (10 μM concentration) of primer AGCTAGCTGCGGACCCGGCGGGGAGGaG and 1 μL of the reverse primer GGCTGCAGCCCTCACCACAACCC. The lowercase letter in the forward primer indicates a mismatch that introduces a restriction site for the BsrBI enzyme (Thermofischer, Waltham, MA, USA). For the digestion stage, samples were incubated for 2 hours at 37 °C with 3 U of the BrsBI restriction enzyme. After this, the DNA fragments were separated by gel electrophoresis on 3% agarose with SYBR Safe (Applied Biosystems, Foster City, CA, USA). The presence of the C allele was identified by fragments of 28 and 212 base pairs (bp), while the presence of the undigested T allele, represented by a 240 bp fragment (13).

### Serum PON1 Activity

PON1 arylesterase activity was measured through the formation of phenol, as validated before (20). The working reagent consisted of 20 mM Tris/HCl buffer, pH 8.0, including 1 mM CaCl_2_ and 1 mM phenylacetate as a substrate. The samples, before being added to the working reagent, were diluted 1:3 in the buffer without phenylacetate and the change in absorbance was recorded for 60 seconds at 270 nm. One unit of arylesterase activity was considered equal to 1 mM of phenol formed per minute and expressed in U/mL. Blank samples containing only water were used to correct non-enzymatic hydrolysis.

### Statistical analysis

Data was analyzed using SAS University Edition (SAS, Cary, NC, USA). Age and gender were used as co-variates in the analysis. The MIXED procedure was used to test the effect of SNPs on PON1 serum activity. Additionally, serum PON1 activity was classified in 3 percentiles in High, Medium and Low PON1 for comparison of dietary intake among the 3 groups. A stepwise logistic regression procedure was performed to identify independent variables that contributed the most for predicting serum PON1 activity. A backward selection technique was used to eliminate covariates that did not contribute to the model. A significance level of 0.20 or above was used to remove covariates from the multivariable model, and a value of 0.15 or less was used to include variables. A P value ≤ 0.05 was considered statistically significant for an all analysis. Data are presented as mean ± standard error of mean.

## RESULTS

### Effects of the PON1 T(-107)C genotype on serum PON1 activity

The characteristics of sample are showed in Table 1. There were no genotype differences for major factors. It is important to note that all volunteers in this study were under medical supervision and were properly medicated. Even so, the systolic blood pressure was lower for CT individuals (p<0.05). The genotype distribution was in Hardy-Weinberg equilibrium (P=0.79).

**Table 1:** Main parameters according to the PON1 T(-107)C genotype of patients with cardiovascular disease under secondary prevention

	Overall	CC	CT	TT	p value^1^
General characteristics					
Number of patients	64	13 (18%)	36 (51%)	15 (30%)	
Sex (% of men)	43 (67.1%)	8 (61.5%)	23 (63%)	12 (80%)	> 0.05
Age (years)	60.5 ± 1.1	57.3 ± 2.5	62.5 ± 1.5	58.2 ± 2.3	0.11
BMI (kg/m^2^)	28.5 ± 4.3	28.7 ± 1.2	28.0 ± 0.7	29.3 ± 1.1	0.62
Waist circumference (cm)	98.5 ± 10.7	96.3 ± 3.0	98.5 ± 1.8	100.5 ± 2.8	0.60
Systolic pressure (mmHg)	130.5 ± 20.9	118.3 ± 5.6	135.7 ± 3.3	128.0 ± 5.2	0.03
Diastolic pressure (mmHg)	79.5 ± 11.1	76.3 ± 3.2	81.6 ± 1.9	78.2 ± 3.0	0.33
Cholesterol (mg/dL)	160.4 ± 40.2	153.9 ± 11.4	161.6 ± 6.8	161.8 ± 10.6	0.83
LDL (mg/dL)	90.6 ± 37.9	86.9 ± 10.7	91.4 ± 6.4	91.5 ± 9.9	0.93
HDL (mg/dL)	37.8 ± 10.7	39.6 ± 3.0	38.6 ± 1.8	32.9 ± 2.8	0.19
Triacylglycerides (mg/dL)	160.6 ± 85.3	147.3 ± 23.6	161.1 ± 14.1	176.93± 21.9	0.65
Glucose (mg/dl)	125.6 ± 53.2	104.6 ± 14.5	134.5 ± 8.7	124.5 ± 13.5	0.21
Caloric intake (kcal)	1323.7 ± 504.5	1604.5 ± 145.5	1319.2 ± 87.4	1278.2 ± 135.4	0.19
Carbohydrates (g)	172.3 ± 66.7	183.7 ± 19.5	178.6 ± 11.7	160.3 ± 18.2	0.62
Protein (g)	63.9 ± 32.1	79.5 ± 9.2	61.3 ± 5.5	61.4 ± 8.5	0.22
Fat (g)	43.9 ± 27.9	63.3 ± 7.8	41.8 ± 4.7	45.2 ± 7.2	0.06

1 MIXED procedure followed by the Turkey post-hoc test using gender and BMI as co-variates.

**Figure 1:**
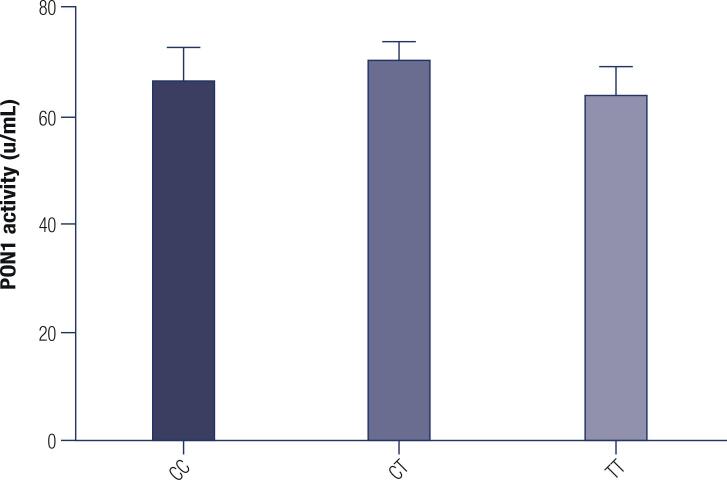
Serum PON1 activity among PON1 T(-107)C genotypes.

Serum PON1 activity was not different between PON1 T(-107)C genotypes as showed in Figure 1 (p>0.05).

### Effect of dietary intake on PON1 activity

Regarding dietary factors showed in Table 2, we observed that intake of cholesterol, SFA and monounsaturated fatty acids (MUFAS) was higher in patients with lower PON1 activity. Lipid ingestion tended (p=0.08) to be higher in patients with lower PON1 activity.

**Table 2:** Dietary nutrient intake according to serum paraoxonase 1 (PON1) activity

	Low PON1	Medium PON1	High PON1	P Value^1^
Energy intake (kcal)	1506.9 ± 112.6	1333.7 ± 115.2	1255.3 ± 115.2	0.28
Protein (g)	74.5 ± 7.1	58.7 ± 7.2	61.6 ± 7.2	0.26
Carbohydrate (g)	176.4 ± 15.0	182.3 ± 15.4	167.3 ± 15.4	0.78
Total lipids (g)	57.8 ± 6.0	43.0 ± 6.1	39.5 ± 6.1	0.08
Fiber (mg)	17.7 ± 2.6	22.9 ± 2.7	17.7 ± 2.7	0.30
PUFA^2^ (mg)	10.6 ± 1.0	9.1 ± 1.1	8.8 ± 1.1	0.43
MUFA^3^ (mg)	17.4 ± 1.7	12.0 ± 1.7	11.9 ± 1.7	0.04
SFA^4^ (mg)	20.3 ± 2.2	13.8 ± 2.2	13.0 ± 2.2	0.05
Cholesterol (mg)	263.3 ± 31.8	145.2 ± 33.4	193.0 ± 33.4	0.04
Trans fat (mg)	0.0 ± 0.0	0.0 ± 0.0	0.0 ± 0.0	0.49
Sodium (mg)	2938. 9 ± 257.2	2764.7 ± 263.3	2323.6 ± 263.3	0.23
Calcium (mg)	525.4 ± 82.7	468.7 ± 84.6	479.0 ± 84.6	0.87
Iron (mg)	8.5 ± 0.7	6.8 ± 0.7	6.4 ± 0.7	0.30
Potassium (mg)	2209.1 ± 183.3	1757.0 ± 187.6	1889.8 ± 187.6	0.21
Magnesium (mg)	161.3 ± 15.7	173.8 ± 16.0	161.3 ± 16.0	0.47
Phosphor (mg)	935.5 ± 72.7	734.4 ± 74.5	777.7 ± 74.5	0.13
Copper (ug)	3.5 ± 2.3	0.9 ± 2.4	4.9 ± 2.4	0.50
Zinc (mg)	10.5 ± 1.1	8.3 ± 1.2	8.2 ± 1.2	0.30

1 MIXED procedure followed by the Turkey post-hoc test using gender and BMI as co-variates

2 PUFA (Polyunsaturated Fatty Acids).

3 MUFA (Monounsaturated Fatty Acids).

4 SFA (Saturated Fatty Acids).

### Combined dietary intake and genetics effects on PON1 activity

The multivariate logistic regression model was used to identify which variables contributed the most to the serum PON1 activity in this group of CVD patients (Table 3). Only SFA intake (P=0.03), genotype (P=0.09), gender (P=0.04), age (P=0.07), carbohydrate intake 3 (P=0.16) remained in the final model in this order, respectively.

**Table 3:** Multivariate logistic regression analysis for dietary intake and genetics effects on serum PON1 activity

Effect	Entry Order^1^	Score^2^	P Value	Overall model assessment	
SFA^3^	1	4.5	0.03	Concordant, %	76.9
Genotype	2	4.6	0.09	Discordant, %	22.9
Sex	3	4.1	0.04	Tied, %	0.2
Age	4	3.2	0.07		
Cho intake^4^	5	1.9	0.16		

1 Order for inclusion of the parameter in the model.

2 Chi-square score.

3 Saturated fatty acids.

4 Carbohydrate intake.

## DISCUSSION

In the current study with subjects in secondary prevention for CVD, we observed that the PON1 T(107)C genotype had no effect on serum PON1 activity. In these patients, factors that were most associated with serum reduced serum PON1 activity were the high intake of cholesterol, SFA and MUFA. SFA, genotype, gender, age and carbohydrate intake were the factors contributing the most for variations in serum PON1 activity in the multivariate regression model.

The genotype distribution for the PON1 T(-107)C polymorphism observed in our study (18%:51%:30%) was different from the reported for the American (31%:48%:20%) and world population (45%:39%:15%), for the CC, CT and TT genotypes, respectively (21). Additionally, a previous study in the same city showed different allelic proportion (25%:44%:32%), from a sample composed healthy women (12). The lower incidence of the C allele in our subpopulation can be explained as our population was composed of patients with CVD. The presence of C allele is associated with higher serum PON1 activity and lower CVD risk (22,23), as our sample was formed by CVD subjects, less CC individuals should be expected. Similarly, others found increased incidence of the TT genotype and lower PON1 activity in CVD patients (24). The PCR and enzymatic digestion technique are inexpensive and can be easily be applied in a clinical setting. Some reports suggest that the error rate of this technique can range from 0.1-0.5% (25). Therefore, patient identification can be very precise using this technique.

Interestingly, in the present study we observed no associations between PON1 C(-107)T genotypes and serum enzyme activity. Similarly, no differences in PON1 activity between genotypes was reported in subjects with diabetes (26). This suggests that other factors besides genetics are important in determining serum PON1 activity in CVD patients. It is important to emphasize that the literature has no studies comparing the effect PON1 C(-107)T genotypes on serum PON1 activity in CVD patients. Additionally, evidence suggests an important role of inflammation in PON1 activity, as PON1 activity is reduced in inflammatory diseases, predisposing LDL to oxidation and exacerbating the atheromatous lesion (27).

Literature shows that 12.6% of serum PON1 variation is predicted by the polymorphism C (-107) T SNP located in the PON1 promoter gene, followed by the consumption of cholesterol (10). We observed that genetics had no effect on PON1 activity in CVD patients. However, the intake of MUFA, SFA and cholesterol had a marked effect. Consumption of SFA has neutral or lowering effects on HDL concentrations (28,29), thus increasing the risk of CVD (29). Likewise, serum PON1 activity is also reduced by the consumption of fats in diet (30–32), since PON1 and HDL levels are correlated (5). Thus, our study in CVD patients is in line with previous studies in the healthy population, where the consumption of these dietary factors predisposes to a reduction in serum PON1 and an increased risk of CVD. The total consumption of lipids in our study tended to be higher in individuals with lower PON1 activity. This variable may have been influenced by the low-fat consumption adopted by our sample, which was composed of people who have already suffered from CVD.

Our results indicate a negative relationship between higher cholesterol consumption and PON1 activity, similarly to that found in mice susceptible to atherosclerosis (32). Cholesterol consumption contributes to about 5.5% of variation in PON1 activity levels (10). These findings diverge from two other studies that show a positive relationship between cholesterol consumption and increased enzyme activity, one seen in baboons (33) and the other in humans (10). Although paradoxical, since PON1 activity is protective, while dietary cholesterol intake is atherogenic, dietary cholesterol is positively and significantly associated with PON1 activity and HDL concentration, thus indicating that the increase in PON1 activity exceeds any increase in HDL (10). Usually cholesterol-rich diets are also abundant in saturated and trans-fat, and even low in PUFAS, which could justify our findings of lower PON1 activity with higher cholesterol consumption.

Our data indicate that the higher intake of SFA, prevalent in the Western diet (34), is negatively associated to serum PON1 activity. Although other authors have not found a direct association between SFA and cholesterol consumption with enzymatic reduction of PON1(35). It is known that the high and prolonged consumption of SFA is associated with an increased risk of CVD (36) for reducing HDL levels (29) to which PON1 is associated (5), corroborating with our findings. Furthermore, a study that evaluated the interaction of the genotype with fat consumption demonstrated that women of the PON-107 CC genotype, ingesting more than 40% of fat from SFA had a significant reduction in serum PON1 activity, although no difference was observed in women of the CT and TT genotypes (11).

Paradoxically to what was expected regarding the effects of MUFAS, our findings indicated a negative relationship between high consumption of MUFA and PON1 activity. One study evaluated that the high intake of oleic acid by men homozygous for the R allele in the PON1-192 polymorphism resulted in increased HDL levels and PON1 activity (37). The consumption of olive oil, rich in oleic acid, after heat treatment by diabetic patients has demonstrated effectiveness in raising postprandial PON1 activity, most notably in women (38). Therefore, in our sample group of CVD patients, in which the greater consumption of MUFAS also coincided with that of other fats can explain these differences. As our patients did not follow a Mediterranean style diet, rich in MUFAs along antioxidant compounds, which can modulate PON1 activity (39,40). In our study with CVD patients, the intake of PUFAs did not seem to be associated with PON1 activity.

The multiple logistic regression analysis showed that the most important factors associated to serum PON1 activity were SFA intake, genotype, gender, age and carbohydrate intake, in this order respectively. Although some individual effects of genotype, gender and age were not observed, their combined interaction can significantly affect PON1 activity. Regarding gender differences, serum PON1 activity in females is higher than in males (41), as estradiol has been shown to enhance PON1 activity independent of liver synthesis (42). Despite this, our previous study showed no difference between pre and post-menopausal women regarding serum PON1 activity (12). The occurrence of CVD in premenopausal women is lower than in men of the same age, however, it increases in postmenopausal women to levels comparable to men (43), due atherosclerotic plaque formation being slower in women (44). Furthermore, the multiple regression model indicated that carbohydrate intake had a small contribution to serum PON1 activity in these CVD patients. One study found association between serum PON1 activity and carbohydrate intake, but just in subjects with cardiovascular risk factors, with no association in CVD group (45). Total carbohydrate intake was inversely associated with HDL cholesterol concentrations (44), which could in turn affect negatively PON1 activity as we observed in this work.

There are some limitations in our study. Recall was performed once, which may not reflect habitual consumption and presents a memory bias and also the low number of individual for a genotype study. Nevertheless, this study showed, for the first time, the consumption of SFA, MUFA and carbohydrate, which can be modified through nutritional intervention, associated with serum PON1 activity, an important enzyme involved in cardiovascular health.

In this sample of patients with CVD, high intake of cholesterol, SFA and MUFA were associated with reduced serum PON1 activity, while PON1 T(1-07)C genotype had no effect. The multiple logistic regression model indicated that SFA, genotype, gender, age and carbohydrate intake were the factors contributing the most for variations in serum PON1 activity. Based on these findings, nutritional guidance for these patients becomes essential, since dietary components interact with serum PON1 activity more than genetic factors.
